# Post-operative volumes following endoscopic surgery for non-functioning pituitary macroadenomas are predictive of further intervention, but not endocrine outcomes

**DOI:** 10.1186/s12902-021-00777-8

**Published:** 2021-06-10

**Authors:** K. Seejore, S. A. Alavi, S. M. Pearson, J. M. W. Robins, B. Alromhain, A. Sheikh, P. Nix, T. Wilson, S. M. Orme, A. Tyagi, N. Phillips, R. D. Murray

**Affiliations:** 1 Leeds Centre for Diabetes & Endocrinology, St James’s University Hospital, Leeds Teaching Hospitals NHS Trust, Leeds, UK; 2 Leeds Institute of Cardiovascular and Metabolic Medicine, University of Leeds, Leeds, UK; 3 Department of Neurosurgery, Leeds Centre for Neurosciences, Leeds Teaching Hospitals NHS Trust, Leeds, UK; 4 Department of Ear, Nose and Throat (ENT) Surgery, Leeds Teaching Hospitals NHS Trust, Leeds, UK

**Keywords:** Non-functioning pituitary macroadenomas, Transsphenoidal surgery, Endocrine function, Tumour volume

## Abstract

**Background:**

Transsphenoidal surgery (TSS) remains the treatment of choice for non-functioning pituitary macroadenomas (NFPMA). The value of measuring tumour volumes before and after surgery, and its influence on endocrine outcomes and further treatment of the residual or recurrent tumour are unknown.

**Methods:**

Data from patients who underwent endoscopic TSS for a NFPMA (2009–2018) in a UK tertiary centre were analysed for pre- and post-operative endocrine and surgical outcomes.

**Results:**

Of 173 patients with NFPMA, 159 (61% male) were treatment naïve. At presentation, 76.2% (77/101) had ≥1 pituitary axis deficit. Older age (*p* = 0.002) was an independent predictor for multiple hormonal deficiencies. Preoperative tumour volume did not correlate with degree of hypopituitarism. Postoperative tumour volume and extent of tumour resection were not predictive of new onset hypopituitarism. Hormonal recovery was observed in 16 patients (20.8%) with impaired pituitary function, with the greatest recovery in the hypothalamic-pituitary-adrenal axis (21.2%, 7/33). A larger residual tumour volume was predictive of adjuvant radiotherapy (3.40 vs. 1.24 cm^3^, *p* = 0.005) and likelihood for repeat surgery (5.40 vs. 1.67cm^3^, *p* = 0.004).

**Conclusion:**

Pre- and post-operative NFPMA volumes fail to predict the number of pituitary hormone deficits, however, greater post-operative residual volumes increase the likelihood of further intervention to control tumour growth.

## Background

Non-functioning pituitary adenomas (NFPA) are benign tumours that constitute around one-third of all pituitary neoplasms, have a prevalence of 7–22 cases per 100,000 population [1, 2] and a standardized incidence rate of 1.02–1.08/100,000 [3, 4]. As they do not cause pituitary hormonal hypersecretion syndromes, the diagnosis is either made incidentally during radiological imaging for other indications or when they are large enough to exert pressure effects to surrounding tissues, resulting in headaches and / or visual defects from optic chiasm compression [5]. Notably, in the majority of patients pituitary hormone deficits are demonstrable at presentation. Studies assessing endocrine function in these individuals using various diagnostic criteria have shown growth hormone (GH) deficiency to be present in 77–88% of patients at presentation, gonadotropin (Gn) deficiency in 61–78%, while corticotropin (19–53%) and thyrotropin (19–43%) deficiencies are noted to a lesser degree [6–10].

NFPA are categorized as macroadenomas when larger than 10 mm in diameter. The treatment of choice in patients with non-functioning pituitary macroadenomas (NFPMA) is transsphenoidal surgery (TSS), aiming for preservation or restoration of vision and long-term tumour control. More recently, the endoscopic endonasal technique is emerging as the primary choice for surgical debulking as an alternative to the microscopic approach [11–13]. Regardless of either technique, surgery should only be performed by an experienced dedicated pituitary surgeon as this enhances success rate and reduces complications [6]. Nonetheless, hypopituitarism prevails in a considerable portion of patients following surgery and is expected to worsen if adjuvant pituitary radiotherapy is required.

In the present study we analysed results of a consecutive series of patients who underwent TSS for resection of NFPMA since adoption of the endoscopic approach in our centre in July 2009. The primary aim was to examine the predictive value of pre-operative tumour volume and degree of adenoma resection on endocrine and tumoural outcomes.

## Methods

### Subjects

We performed a retrospective analysis of consecutive adult patients who underwent endoscopic transsphenoidal resection for non-functioning pituitary adenoma between 01 July 2009 and 31 August 2018 at Leeds Teaching Hospitals NHS Trust (LTHT). Only macroadenomas were included.

Patients were identified using the neurosurgical database collated at the time of surgery by the pituitary surgeons (neurosurgery and ear, nose and throat) within our institution. LTHT is a dedicated multidisciplinary centre of pituitary expertise in the North and West Yorkshire region of the UK, serving a population of 2.9 million [14]. Patients were referred to the neurosurgical service mainly from the endocrinology department at LTHT and also directly from various hospitals in the catchment area (including six peripheral hospitals) after initial endocrine evaluation. Details concerning therapeutic intervention and results of endocrine investigations were collected from patients’ medical notes and electronic patient records (PATIENT PATHWAY MANAGER and the PATHOLOGY RESULTS SERVER). Data were compiled by trained staff over a four-month period between December 2018 and March 2019.

Patient episodes were subdivided into those which were performed in patients who were surgically naïve (Group A), and those representing second surgery for their pituitary adenoma (repeat endoscopic TSS, or endoscopic TSS following previous microscopic TSS / transcranial tumour resection; Group B). For each patient, information at baseline, and after surgery were recorded – namely at the first post-operative endocrine evaluation (usually 6 weeks) and at the time of the last clinic assessment. These included demographic details, surgical and radiology reports, tumour histology, pre-operative and post-operative tumour volume, endocrine assessments as well as the need for radiotherapy and further surgery.

### Evaluation of pituitary function

Pituitary function testing was performed at baseline and at 6 weeks postoperatively. The presence of hypopituitarism (partial or complete), number of axes involved, the presence of diabetes insipidus, and hyperprolactinaemia were registered.

#### GH and Hypothalamo–Pituitary–Adrenal (HPA) axes

The insulin tolerance test (ITT) was the preferred method for assessing the integrity of the GH and HPA axes. Where the ITT was contraindicated (i.e. history of epilepsy, increased cardiovascular risk, age > 65 years), the glucagon stimulation test (GST) was performed [15]. Pituitary investigations were performed at presentation and 6 weeks postoperatively. Patients who underwent radiotherapy (RT) underwent a further dynamic pituitary test in the first year following treatment and annually afterwards unless GH and ACTH deficiency had previously been confirmed. Both for the ITT and the GST, severe GH deficiency was defined as a peak GH response of less than 3 mcg/l [15]. Patients with incomplete pre- or post-operative GH dynamic testing were excluded in the final analysis for endocrine evaluation. GH status at baseline was not routinely assessed by stimulation test in the regional peripheral hospitals. Additionally, where urgent debulking was necessary (apoplexy, acute visual deterioration), dynamic GH testing was not prioritised in the preoperative setting. ACTH deficiency in our laboratory was defined by a locally validated peak cortisol of less than 500 nmol/l for the ITT and less than 450 nmol/l for the GST [15]. Patients with borderline cortisol response to the GST (peak cortisol between 400 and 450 nmol/l) were subsequently investigated using the short Synacthen test (SST), to determine the clinical need for glucocorticoid replacement therapy [15]. Cortisol values at baseline (09:00 h) and 30 min following 250 μg of intramuscular cosyntropin (tetracosactide) were used to define adrenal insufficiency. The cut-off for an acceptable response was defined as greater than 450 nmol/l, based on local normative data.

#### Additional anterior pituitary hormones

LH, FSH, TSH, prolactin, IGF-1, free T4, testosterone in males and oestradiol in females, were measured in morning serum samples [15]. In males, hypogonadotrophic hypogonadism was defined by a low serum testosterone (< 8 nmol/l) in combination with inappropriately normal or low gonadotropins (Gn) [15]. In premenopausal females, Gn deficiency was defined as the presence of symptoms of oligo- or amenorrhoea, low oestradiol levels and inappropriately normal or low LH/FSH [15]. In postmenopausal women, the absence of elevated Gn concentration (< 30 IU/L) was indicative of Gn deficiency [15]. Low levels of free thyroxine (fT4; normal range 10–20 pmol/l) in the presence of low, normal or mildly elevated TSH (<6miu/l), in patients without a pre-existing history of thyroid disease, were deemed diagnostic of thyrotroph dysfunction [15]. Permanent diabetes insipidus (DI) was diagnosed as the persistence of hypotonic polyuria, responsive to desmopressin (DDAVP) at the 6-week postoperative assessment; water deprivation tests were not routinely performed. Multiple hormone deficiencies were defined by deficiency of at least two individual anterior pituitary hormone axes. Panhypopituitarism was defined as the co-existence of GH, Gn, ACTH and TSH deficiencies. Patients lacking laboratory values for a particular axis were excluded from the analysis of that particular axis.

After the initial postoperative endocrine assessment, patients were reviewed on an annual basis during follow-up and subsequent hormone testing was carried out at the discretion of the clinician. Patients routinely had an annual pituitary hormone profile to assess the adequacy of hormone replacement. Dynamic testing of the GH and HPA axes were performed post-radiotherapy or during tumour recurrence/ growth as indicated.

#### Laboratory assays

GH and IGF-1 were measured using SIEMENS IMMUNLITE 2000 methodology (GH calibrated against WHO NIBSC IS 98/574) [15]. Cortisol, prolactin and oestradiol were measured by immunoassay on the SIEMENS ADVIA CENTAUR. LH, FSH, TSH and free T4 were measured by chemiluminescence using SIEMENS ADVIA CENTAUR and testosterone was measured using isotope-dilution liquid chromatography–tandem mass spectrometry [15]. All assays were performed in the routine clinical biochemistry laboratories within the Leeds Teaching Hospitals NHS Trust and have been regularly validated by internal quality control (IQC) and external quality assessment (EQA) [15].

#### Imaging study and tumour volume

The radiographic evaluation consisted of 1.5 T magnetic resonance imaging (MRI) with and without contrast performed pre-operatively, at 3–6 months post-surgery and yearly thereafter for the first 5 years.

Tumour volume was measured using MRI with BRAINLAB IPLAN NET software pre-operatively and post-operatively at 3 months after surgery. Gross total resection (GTR) was achieved when complete macroscopic removal of the adenoma had been carried out (defined as absence of visible tumour residuum on post-operative MRI as reported by the neuroradiologist); near-total resection (NTR) when more than 90% of the tumour was removed; sub-total resection (STR) when 75 to 90% of the tumour was removed; and partial resection when less than 75% of the tumour was removed [16]. Extent of resection (EOR) was calculated based on volumetric analysis of pre-and post-operative contrast-enhanced MRI according to the formula: [(Preoperative tumour volume (PTV) – Residual tumour volume) / PTV] × 100%.

#### Follow up

All patients had a post-operative MRI scan at 3 months after TSS. This was followed by annual neurosurveillance imaging for the next 5 years and 2-yearly thereafter, unless patients became symptomatic of headaches or visual loss which would warrant earlier imaging outside the surveillance protocol. Tumour recurrence was defined by the appearance of visible tumour on pituitary imaging after GTR had been previously achieved. Tumour regrowth was defined by enlargement of a remnant tumour after TSS following review by a dedicated pituitary neuroradiologist. Patients with remnant tumours or recurrence in proximity to the optic chiasm had annual visual field assessment as part of monitoring. Further intervention, either in the form of repeat surgery or radiotherapy, was considered on a case-by-case basis after discussion with neurosurgeons and oncologists in a multidisciplinary setting.

#### Statistical analysis

Results are presented as mean and standard deviation, or median and range for parametric and nonparametric data respectively [15]. Chi-squared and Fisher exact tests were used to compare frequencies and proportions of categorical variables. The Mann–Whitney U-test and student’s t-tests were used to compare differences between continuous variables (age, tumour volume, extent of resection) in patient groups with different endocrine outcomes (new axis deficit vs. preserved pituitary function). A multiple linear regression analysis was performed to examine variables contributing to the development of pituitary hormone deficits. A *p*-value of < 0.05 was considered statistically significant for all tests. The regrowth-free curves were generated by the Kaplan-Meier method. Statistical analysis was performed using MICROSOFT EXCEL (Version 16.43) and GRAPHPAD PRISM 8 [Statistical software, Version 8.4.3 (471)].

#### Ethical considerations

This study was designed as a clinical service review to assess current practice, make quality improvements and improve patient outcomes. Given that it was undertaken using retrospective de-identified data collected during the course of routine care by the direct care team as part of usual practice, no informed consent was requested from the patients. The study protocol was approved by the local Research and Innovation department at Leeds Teaching Hospitals NHS Trust who acknowledged waiver of consent and confirmed that the study did not require Health Research Authority (HRA) approval and therefore no NHS Research Ethics Committee (REC) review. Research data for this study was obtained from the neurosurgical database created and maintained by the local pituitary surgeons at LTHT (neurosurgeons NP and AT and ENT surgeons PN and TW) as part of routine postoperative practice. No administrative permissions were required to access this anonymized data provided by co-authors NP, AT, PN and TW who shared ownership rights.

All methods were carried out in accordance with relevant guidelines and regulations for medical research involving human subjects of the Declaration of Helsinki.

## Results

### Patient characteristics

A total of 173 patients with NFPMA who underwent endoscopic endonasal TSS were identified (107 males and 66 females). Of these individuals 159 were treatment naïve (Group A), and 14 had undergone previous surgery via microscopic TSS or transcranial surgery – 1/14 had received radiotherapy after the first surgery. Of the treatment naïve patients (Group A) 9/159 went on to have further surgery during the follow-up period, none of whom underwent prior radiotherapy. Therefore, data were available on a total of 23 patients who underwent endoscopic TSS, having had prior pituitary surgery (Group B).

### Treatment Naïve patients (group a; Table 1)

The most frequent presentation was visual loss (*n* = 90/159, 56.6%). Twenty-seven patients (17.0%) were asymptomatic and the diagnosis of a NFPMA was incidental. Presence of a visual field defect on clinical assessment (*n* = 104, 65.6%) was the most common indication for surgery. The mean patient age at the time of surgery was 59.3 ± 13.3 (range 21–87) years. No significant differences in the age distribution of patients according to gender were found. Eight patients (5.0%) presented acutely, necessitating emergency surgical debulking. Immunohistochemical analysis identified most resected tumours to be silent gonadotroph (*n* = 84, 52.8%) adenomas. Mean length of follow-up post-TSS was 3.1 ± 2.1 years. Eleven deaths were recorded during the study period – three patients died within the first year following TSS, none of which was directly related to the pituitary surgery.

**Table 1:** Characteristics and post-operative outcomes of surgical naïve patients (Group A) undergoing endoscopic TSS

Characteristics	Values	
N	159	
Gender	M: 97 (61.0%); F: 62 (39.0%)	
Mean age at surgery (years)	59.3 ± 13.3; Range = 21-87 years	
**Presentation**		
• Loss of vision	90 (56.6%)	
• Incidental (Asymptomatic)	27 (17.0%)	
• Symptoms of hypopituitarism	18 (11.3%); (8-Hypogonadism)	
• Headaches	13 (8.2%)	
• Apoplexy	9 (5.7%)	
• CN palsy	2 (1.3%)	
Emergency	8 (6- apoplexy, 1-visual loss, 1-CN palsy)	
**Indication for surgery**		
• Visual field defect	104 (65.4%)	
• Contact with optic chiasm/ suprasellar extension (sight-threatening)	33 (20.8%)	
• Apoplexy	9 (5.7%)	
• Large tumour volume	6 (3.8%)	
• Enlarging tumour on surveillance	4 (2.5%)	
• CN involvement	3 (1.9%)	
**Immunohistochemistry**		
• Gonadotroph	84 (52.8%)	
• Null Cell	52 (32.7%)	
• Plurihormonal adenoma	3 (1.9%)	
• Silent corticotroph adenoma	8 (5.0%)	
• Silent thyrotroph adenoma	1 (0.6%)	
• Silent somatotroph adenoma	1 (0.6%)	
• Silent lactotroph adenoma	1 (0.6%)	
• Hyperplasia	1 (0.6%)	
• Other (necrotic, infarcted, normal, no tissue)	8 (5.0%)	
**Post-surgical outcomes**^**a**^		
*Endocrinopathies*	*At diagnosis (n=101)*	*Post-operative (n=101)*
• Normal pituitary function	24 (23.8%)	20 (19.8%)
• 1-2 anterior pituitary axis deficits	41 (40.6%)	43 (42.6%)
• ≥ 3 anterior pituitary axis deficits	36 (35.6%)	38 (37.6%)
• ADH deficiency	0	2 (2.0%) + 2 transient DI
*Individual pituitary axis*	*At diagnosis (n=101)*	*Post-operative (n=101)*
• GH deficiency	60 (59.4%)	71 (70.3%)
• LH/ FSH deficiency	55 (54.5%)	56 (55.4%)
• ACTH deficiency	33 (32.7%)	37 (36.6%)
• TSH deficiency	36 (35.6%)	37 (36.6%)
Preserved pituitary function (*n*=101, total cohort)	60 (59.4%)	
Pituitary axis recovery (*n*=77, excluding 24 patients with normal pituitary function pre-op)	16 (20.8%)	
New pituitary axis dysfunction (only including patients with 0-3 anterior pituitary axes impaired pre-op, *n*=83)	25 (30.1%)	
**Tumour size**		
Median tumour volume (cm^*3*^*)*	*At diagnosis (n=156)*	*Post-operative (n=151)*
	5.51 IQR: 3.52 – 9.48	0.92 IQR: 0.44 – 2.02
Extent of tumour resection (EOR), median (%), n=151	82.3%, IQR: 64.8 – 91.5%	
Gross Total Resection (GTR)	9 (6.0%)	
Near-Total Resection (≥90%)	44 (29.1%), including 9 GTR	
Subtotal Resection (75-89.9%)	53 (35.1%)	
Partial Resection (<75%)	54 (35.8%)	
**Long-term follow-up**		
Mean Follow-up (years)	3.1 ± 2.1 years; Range 0.5-9.6 years	
Large tumour remnant, requiring intervention	14 (8.8%)	
Tumour regrowth	28 (17.6%), of which 24 (15.1%) required intervention	
*Further Treatment*		
• Radiotherapy (RT)	35 (22.0%) External beam RT: 25 (15.7%) Stereotactic radiosurgery: 10 (6.3%)	
• Repeat Surgery	9 (5.7%)	
Deaths	11 (6.9%)	

^a^including only patients with complete formal pre- and post-operative endocrine assessments (i.e. excluding 48 patients without dynamic GH stimulation tests at baseline and/or after surgery and 10 additional patients with other missing endocrine data)

#### Endocrine evaluation

The prevalence rates of GH, Gn, ACTH and TSH deficiencies are shown in Table 1. We compared pre- and post-operative pituitary function only in those patients who had formal pre- and post-operative assessments. Forty-eight patients did not have complete dynamic GH testing either at baseline or after surgery and were excluded from the final analysis for endocrine evaluation. Provocative testing of the GH axis was routinely performed only in patients referred directly from the Endocrinology Department at Leeds Teaching Hospitals, except in presentations requiring urgent tumour debulking. Peripheral hospitals carried out serum IGF-1 measurements, unless patients developed symptoms suggestive of GH deficiency despite receiving optimal treatment for other pituitary hormone deficiencies as necessary. In addition, patients with missing endocrine data pertaining to gonadal, thyroidal or HPA axes at baseline and/ or at follow-up (*n* = 10) were also excluded. Complete pre- and post-operative pituitary hormone function was available in 101/159 patients when the database was frozen on 31st March 2019.

#### At presentation

Twenty-four patients (23.8%) had normal pituitary function, 41 (40.6%) had 1–2 anterior pituitary axis deficits, and 36 patients (35.6%) had at least 3-axis deficits of whom 18 patients, predominantly males (*n* = 14, 77.7%) had panhypopituitarism (all 4 anterior axes impaired), as shown in Table 1. None of the patients had cranial diabetes insipidus at diagnosis. The most frequently affected hormonal axis was GH deficiency (*n* = 61, 60.4%) followed by LH/ FSH deficiency (*n* = 55, 54.5%). Table 2 shows the pituitary hormone function in the study population at baseline. Older age (coeff 0.26, *p* = 0.001) was a significant independent predictive factor for multiple pituitary hormone deficiencies at presentation. Men displayed a higher prevalence of GH deficiency (63.4% vs. 43.5%, *p* = 0.01). Secondary ACTH and LH/FSH deficiencies were more common in older patients at presentation (63.2 vs. 57.2 years, *p* = 0.03; 63.5 vs 54.0 years, *p* < 0.01).

**Table 2:** Pituitary hormone function at baseline in the study population (*n* = 101) and association with different parameters

**Number of pituitary hormone deficiencies (at baseline)**	**Gender**		***p*-value**
	**Males (*n*** **= 62)**	**Females (*n*** **= 39)**	
• 0	12 (19.4%)	12 (41.6%)	0.06
• 1–2	24 (38.7%)	17 (43.5%)	
• ≥3	26 (41.9%)	10 (25.6%)	
**Individual hormone axis**			
• GH deficiency	43 (63.4%)	17 (43.5%)	0.01*
• LH/FSH deficiency	37 (59.7%)	18 (21.2%)	0.22
• ACTH deficiency	22 (35.3%)	11 (28.2%)	0.52
• TSH deficiency	24 (38.7%)	12 (30.8%)	0.52
**Number of pituitary hormone deficiencies (at baseline)**	**Age at Surgery (mean ± SD)/ years**		***p*-value**
• 0	54.7 ± 13.0		0.001*
• 1–2	57.7 ± 12.0		
• ≥3	63.8 ± 11.6		
**Individual hormone axis**	**Age at Surgery (mean ± SD)/ years**		***p*-value**
• GH deficiency	60.6 ± 11.6		0.16
• LH/FSH deficiency	63.5 ± 11.0		< 0.01*
• ACTH deficiency	63.2 ± 13.2		0.03*
• TSH deficiency	62.2 ± 12.0		0.06

*p < 0.05 is statistically significant

#### Post-operative outcomes

Overall 20 patients (19.8%) had normal pituitary function post-operatively. Pituitary function remained unchanged postoperatively in 59% (*n* = 60) of patients. New axis deterioration was observed in 25 patients after TSS, corresponding to an overall proportion of 30.1% (25/83) when excluding the patients who already had all 4 anterior axes impaired pre-operatively (*n* = 18) and thus could not have further hormone deficits (Table 1).

**Fig. 1:**
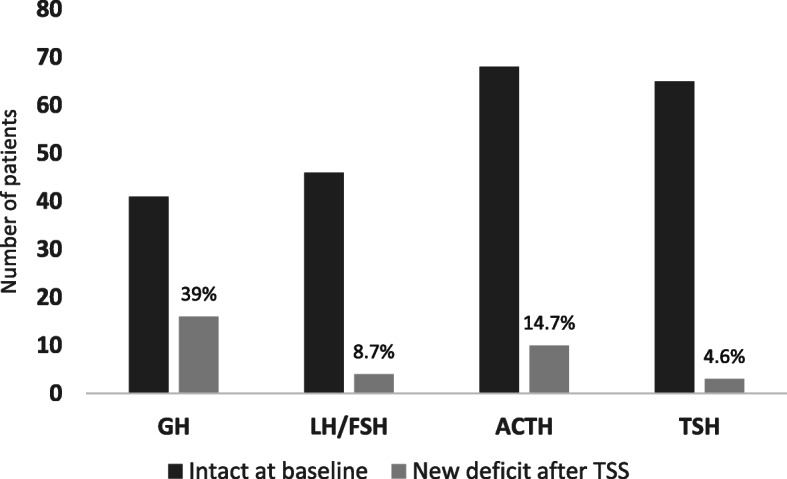
Incidence of new onset pituitary hormone axis deficiency after TSS. For each hormone axis, the first bar denotes the number of patients with normal function at baseline and the second bar denotes the percentage of these patients who developed hormonal deficit after TSS. GH axis was the mostly affected post-operatively (39%) followed by ACTH deficiency (14.7%)

Considering the pituitary axes individually, the GH axis was most susceptible to damage post-operatively. New GH deficiency appeared in 39.0% (*n* = 16/41) of the 41 patients with normal GH function pre-operatively. Figure 1 shows the incidence of new onset anterior pituitary hormone dysfunction for each individual axis.

**Fig. 2:**
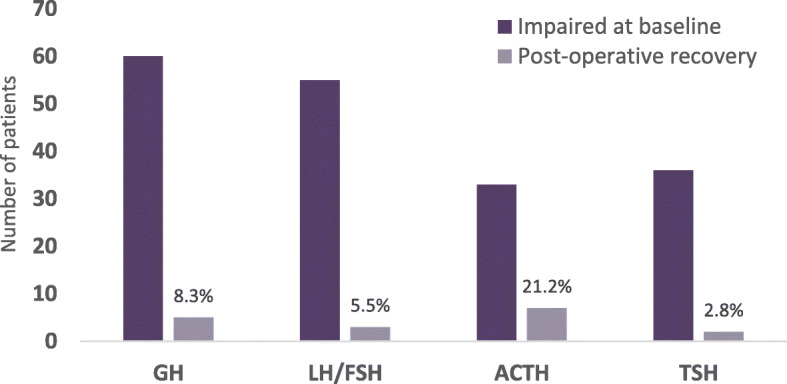
Recovery of pituitary hormone dysfunction after TSS. For each hormonal axis, the first bar denotes the number of patients with that particular hormone deficit at baseline and the second bar shows the proportion of patients who recovered normal function in that axis. The HPA axis showed highest recovery post-operatively at 21.2% followed by GH (8.3%), LH/FSH (5.5%) and TSH (2.8%) respectively

Pituitary axis recovery was noted in 20.8% of patients (16/77 patients, excluding 24 patients who had normal baseline pituitary function pre-operatively). Figure 2 shows the rate of recovery in each individual pituitary axis after TSS. The highest rate of recovery was noted in the HPA axis, where 7/33 (21.2%) with impairment of the axis pre-operatively recovered normal function.

#### Volumetric analysis and tumour resection

The median pre-operative tumour volume was 5.51 cm^3^ (IQR 3.52–9.48 cm^3^) and median post-operative residual tumour volume was 0.92 cm^3^ (IQR 0.44–2.02 cm^3^). In the 151 patients with preoperative and postoperative MRI studies available for volumetric analysis, the median extent of resection (EOR) was 82.3%, (IQR 64.8–91.5%). Table 3 shows the EOR achieved in the study population.

**Table 3:** Tumour size and the impact on endocrine function in the study population (*n* = 101) at baseline and after TSS

**Preoperative tumour volume**	**Males** ***(n*** **= 62)**	**Females (*n*** **= 39)**	***p*-value**
Mean tumour size (cm^3^) ± SD	8.88 ± 8.56	6.46 ± 5.05	0.12
**Age at surgery (years)**	59.3 ± 13.3		0.35
**Individual hormone axis**	**Mean tumour size (cm**^**3**^**)**		***p*-value**
• GH deficiency	8.45 ± 6.10		0.44
• LH/FSH deficiency	8.81 ± 8.92		0.22
• ACTH deficiency	7.17 ± 5.34		0.45
• TSH deficiency	7.88 ± 6.41		0.93
**Number of pituitary hormone deficiencies**			
• 0	6.28 ± 4.18		0.69
• 1–2	9.00 ± 9.88		
• ≥3	7.92 ± 5.91		
**Postoperative Tumour Residuum**			
Mean tumour residuum size (cm^3^) ± SD	1.96 ± 4.46		
**Number of pituitary hormone deficiencies**	**Mean tumour size (cm**^**3**^**)**		***p*-value**
• 0	1.68 ± 2.29		0.44
• 1–2	2.34 ± 6.39		
• ≥ 3	1.24 ± 2.07		
**Extent of Resection (EOR)**			
Mean EOR (%) ± SD	75.9 ± 21.8; Range: 3.4–100%		
Gross total resection (GTR)	7 (6.9%)		
Near total resection (≥90 % )	28 (27.7%), including 7 GTR		
Subtotal resection (75–89.9%)	39 (38.6%)		
Partial resection (< 75%)	30 (29.7%)		
**Endocrine outcomes**	**Mean EOR (%) ± SD**		***p*-value**
• New axis deficit (*n* = 25)	78.6 ± 23.5		0.48
• Axis recovery (*n* = 16)	79.9 ± 16.9		0.45
• Pituitary function preserved (*n* = 60)	73.8 ± 22.1		0.38

The effects of the extent of tumour resection are studied on the appearance of new hormone deficits and on axis recovery. A *p*-value of < 0.05 is considered statistically significant

#### Tumour size and pituitary function

The preoperative tumour volume did not correlate with either gender (8.88 vs. 6.46 cm^3^, *p* = 0.12) or age at the time of surgery (*p* = 0.35). Preoperative tumour size also did not predict the severity of hypopituitarism (number of axes affected) at diagnosis (6.28 vs. 9.00 vs. 7.92 cm^3^, *p* = 0.69). Individual pituitary hormone axis deficiencies at diagnosis were independent of tumour size as shown in Table 3. Likewise, in a multilinear regression, the absolute postoperative volume of residuum did not predict the degree of pituitary dysfunction postoperatively (*p* = 0.44). Most importantly, the EOR (both percentage and absolute volume reduction) during TSS was independent of the new onset of pituitary hormone deficiencies postoperatively (78.6 ± 23.5 vs. 75.0 ± 21.2 cm^3^, *p* = 0.48). Similarly, EOR was also not associated with recovery of pituitary function after surgery (79.9 ± 16.9 vs. 75.2 ± 22.6 cm^3^, *p* = 0.45). The results are summarised in Table 3.

#### Tumour size and further intervention

Table 1 shows the long-term outcomes in the study population at the end of follow-up. After a mean follow-up of 3.1 ± 2.1 years, 38 patients (23.9%) required further intervention (radiation and/ or repeat surgery). 24 (15.1%) of these patients had demonstrated tumour regrowth of the adenoma; the remainder 14 patients had a significant tumour remnant with suprasellar extension visible on scan postoperatively and were empirically treated. Nine patients (5.7%) went on to have repeat surgery. The indication for repeat TSS was tumour regrowth causing visual field loss or threatening sight in eight patients (8/9, 89%). In the remaining patient, the decision for repeat debulking surgery was due to regrowth in a sizeable tumour residuum in a young adult. The mean interval between primary and repeat surgery was 2.4 ± 1.2 years (range: 0.3–4.2 years). Thirty-five (22.0%) patients were submitted to adjuvant radiotherapy (RT), of whom 25 received conventional fractionated external beam irradiation and 10 received stereotactic gamma knife radiosurgery (SRS). Six patients required both repeat surgery followed by adjuvant radiotherapy.

**Fig. 3:**
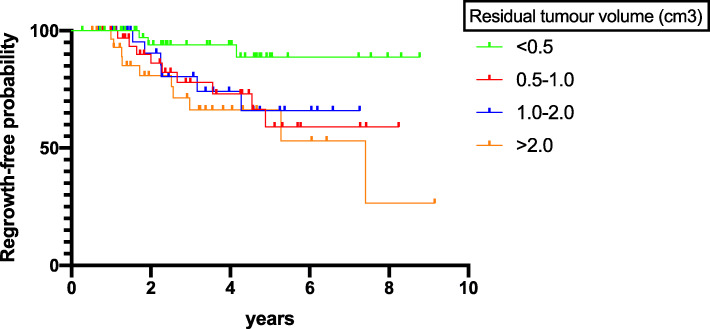
Kaplan-Meier regrowth-free survival curves for total cohort of patients stratified by size of residual tumour volume (cm3). A larger residual tumour volume increased the probability of tumour regrowth during follow-up (*p* = 0.03)

During a mean follow-up of 3.1 years, 28 patients demonstrated a degree of tumour regrowth, including 24 who required further intervention and another 4 who were not subject to secondary treatment during the study period. When analysed by residual tumour volume, it was clear that a larger residuum significantly increased the risk of tumour regrowth during neurosurveillance imaging. This is illustrated in Fig. 3.

A larger residual tumour volume increased the likelihood of further intervention (repeat TSS or RT, *n* = 38) during follow-up (3.88 ± 6.82 vs. 1.24 ± 1.62 cm^3^, *p* < 0.001). When analysed separately, a large residual tumour volume was predictive of adjuvant radiotherapy (3.40 vs. 1.24 cm^3^, *p* = 0.005) as well as likelihood for repeat surgery (5.40 vs. 1.67cm^3^, *p* = 0.004) during follow-up. Gross total resection was achieved in 9 patients, only 1 of whom developed tumour recurrence 20.5 months after TSS. In addition, presence of a large tumour volume at baseline was also predictive of further intervention (repeat TSS or RT) during follow-up (11.52 ± 11.03 vs. 6.48 ± 4.91 cm^3^, *p* = 0.001), irrespective of EOR.

#### Operative experience and tumour resection (Group A)

Between 2009 and 2013, 48 NFPMA were resected by TSS at our institution whilst 111 tumours were debulked during the following 5-year period from 2014 to 2018. The median EOR was 78.3% (IQR 63.5–84.2%) during the first-half of the study period compared with 86.4% (IQR 70.4–93.8%, *p* = 0.005) in the latter half. Despite a net improvement in EOR during the latter 5 years most likely related to improved surgical technique and experience, the incidence of new post-operative pituitary axis dysfunction was not significantly different between the two groups (23.5 vs. 25.4%, *p* = 0.99). Analysing both study periods separately, it transpired that the onset of new endocrine deficits after TSS was independent of the EOR in each cohort (first half: 2009–2013, median EOR 82.7 vs. 76.2%, *p* = 0.15; second half: 2014–2018, median EOR 82.9 vs. 83.9%, *p* = 0.36), highlighting that endocrine outcomes are independent of EOR. The results are illustrated in Table 4.

**Table 4:** Comparison of parameters between the first and second half of the study period

Characteristic	Study period		***p***-value
	2009–2013	2014–2018	
Patients (*n* = 159)	48	111	
Preoperative tumour volume (median, cm^3^)	5.51 (IQR: 2.84–9.56)	5.51 (IQR: 3.56–9.27)	0.75
Postoperative residual tumour volume (median, cm^3^)	1.28 (IQR: 0.61–2.19)	0.77 (IQR: 0.39–1.72)	0.01*
EOR (median, %)	78.3 (IQR: 63.5–84.2)	86.4 (IQR: 70.4–93.8)	0.005**
Endocrine Outcomes (*n* = 101)^a^			
• Preserved function	23/34 (67.6%)	37/67 (56.7%)	0.29
• New hormone deficit(s)	8/34 (23.5%)	17/67 (25.4%)	0.99
• Axis recovery	3/34 (8.8%)	13/67 (19.4%)	0.25

^a^Complete endocrine data pre- and post-TSS was available in 101 patients (34 of whom were in the first half, 2009–2013 and 67 in the second half, 2014–2018)

### Previous surgery cohort (Group B)

Our cohort included 23 patients (16 males; 57.0 ± 12.9 years) who underwent endoscopic TSS having had prior pituitary surgery by microscopic TSS or transcranial route (n = 14) or endoscopic TSS (*n* = 9). One patient had undergone radiotherapy after the first surgery. Comparing patients who had primary surgery (microscopic TSS or transcranial surgery) before the implementation of the endoscopic TSS technique with those who had prior endoscopic TSS surgery, there was no significant difference in EOR rates (median EOR 66.5% vs. 77.1%, *p* = 0.20) after repeat surgery. Unfortunately, we did not have tumour volume data to compute EOR rates on the initial surgery undergone by the 14 patients prior to endoscopic TSS.

On the other hand, although the mean preoperative tumour volume did not significantly differ between patients who had prior surgical intervention before the study period when compared to those who were surgery-naïve, having had previous surgery was predictive of decreased EOR (68.9% [IQR 42.4–80.2%] vs. 82.3% [IQR 64.8–91.5], *p* = 0.017).

## Discussion

We performed a cross-sectional study of consecutive NFPMA treated surgically since adoption of endoscopic transsphenoidal surgery in 2009 at our institution. Comprehensive clinical characterisation was undertaken of endocrine status, pre- and post-operative tumour volumes, and the requirement for further interventions. The most important finding from these data is that pre- and post-operative volumes, as well as EOR did not correlate with the prevalence of pituitary hormone deficits, however, the post-operative residual volume was predictive of requirement for further intervention to control the tumour mass. To our knowledge this is the first description of the impact of pituitary tumour volumes and EOR on both endocrine status and the need for further intervention for the tumour. In support of the dissociation between EOR / post-operative tumour volumes and endocrine status, greater EOR was achieved in the latter half of the study period, however, without change in endocrine outcomes. Arguably, this could also be the result of improved surgical experience, resulting in more accurate resection margins with better visualisation, without further compromise to endocrine function. Interestingly, but not unexpectedly we also showed that in patients who required second surgery the EOR was significantly lower compared with surgical naïve subjects.

In addition to symptoms from mass effect, NFPMA are associated with a variable degree of hypopituitarism in the majority of cases at presentation [6, 7, 17]. In keeping with previously described cohorts of NFPMA, at presentation our cohort showed hypopituitarism in 76.2% [6, 7, 17, 18], with the GH and Gn axes being most frequently impaired [6, 7, 17, 19], and a greater prevalence in those presenting at an older age [19–21]. We could find no correlation between pre-operative adenoma volume and the presence of pituitary hormone deficits. Previous reports have been inconsistent, with reports of no relationship between pre-operative adenoma volume and pituitary hormone deficits [21–23] and other studies showing larger tumours to have a higher prevalence of deficits [19, 24]. Differences likely relate to heterogeneity of the cohorts studied in relation to tumour size and rigour of endocrine testing. A number of studies have included both micro and macroadenomas [24], albeit microadenomas are infrequently associated with hormone deficits thereby potentially explaining the finding of higher prevalence of pituitary deficits with increasing tumour size. In contrast our study included only macroadenomas. Furthermore, other studies have not evaluated pituitary function with stimulation tests, relying simply on basal hormone levels [19]. In our sub-cohort in which we present endocrine outcomes we have fully characterized endocrine status including stimulation tests to determine GH and cortisol status pre- and post-operatively.

In the absence of proven medical therapies that can reliably reduce NFPMA tumour mass, initial management is limited to clinical assessment and neurosurveillance with MRI scanning, or surgical resection of the tumour. Previously published data suggest tumour regrowth rates of 15–50% within 5 years after surgery alone [25, 26]; size of the post-operative tumour remnant being a major determinant of regrowth [25]. Without adjuvant treatment, regrowth rates of 50–60% were observed after subtotal resection and upwards of 20% after gross total resection with increased length of follow-up [26, 27]. Five-year regrowth rates reduce to 2–28% after surgery followed by adjuvant radiotherapy [25–28].

Neurosurgical guidelines recommend resection of symptomatic NFPMA as first-line treatment [29] in symptomatic NFPMA with the aim of relieving symptoms caused by mass effect; with emphasis on decompression of the optic nerve to preserve or improve vision, but also to reduce headaches, and prevent further deterioration of endocrine function as a secondary outcome. However, preservation and restoration of hormonal function are also essential to assessing the outcome of surgery and to the patient’s quality of life. In this setting, ACTH and gonadotropin deficiencies have been associated with increased mortality risk in a large cohort of NFPA patients [30]. Surgical intervention can be effective in normalising pituitary function with a variable reported improvement in degree of hypopituitarism ranging from 2 to 49% [10, 31–33]. Pituitary function was preserved in 59% of our patients, whilst there was an improvement in pituitary dysfunction in 21% of patients post-operatively. In our study, and as reported by others [8, 34], ACTH was the most frequent axis to show recovery in our study. Resection of the pituitary adenoma reduces intrasellar pressure, providing a mechanism by which pituitary hormone recovery can occur post-operatively [22]. However, chronically elevated intrasellar pressure may lead to ischaemic necrosis of anterior pituitary cells and limited potential to regenerate leading to permanent hypopituitarism.

In our cohort, development of new pituitary hormone deficiencies was independent of initial tumour size, postoperative tumour volume and extent of tumour resection. Our findings are consistent with those of Jahangiri et al. (2016) who also pointed out that preoperative tumour size, postoperative tumour size and extent of resection did not predict development of new postoperative endocrine deficits [20]. A further study showed that although pituitary tumour size did not correlate with postoperative recovery of hypopituitarism [12], absence of tumour residuum on postoperative pituitary imaging correlated with a higher chance of postoperative pituitary function recovery in a mixed cohort of non-functioning and hypersecreting pituitary adenomas. In contrast to these studies others have shown preoperative tumour size to relate to postoperative hormonal deficits [21].

Finally, in our study the presence of a large tumour residuum was predictive of likelihood of further intervention, with adjuvant radiotherapy and/or repeat surgery. This finding is in agreement with the majority of the literature which shows that the volume of postoperative tumour remnant predicts recurrence / progression of the tumour [6, 7, 18, 35], in particular a postoperative tumour remnant with extrasellar component [25]. Postoperative residuum is not unique as a predictor of recurrence / progression, with a number of additional factors being important in this including age, invasiveness, proliferative character of the tumour, and duration of follow-up [35]. Preoperative size has also been observed to correlate with the need for further intervention [36], as evident in our study. Many of these variables are not mutually exclusive as larger tumours preoperatively are more likely to be invasive, and lead to a larger postoperative residuum. The EOR is another crucial parameter in transsphenoidal pituitary surgery and is linked to postoperative morbidity and mortality [37]. In our study, a lower rate of EOR was predictive of adjuvant radiotherapy or repeat TSS during follow-up. Maximising EOR is thus of particular relevance; however several factors, including invasion into the cavernous sinus, graded with the Knosp scale as well as the pituitary adenoma volume, influence the likelihood of achieving gross total resection rates [37]. In our study, insufficient information on the Knosp grade did not allow a robust explanation as to the GTR observed, although a negative association was demonstrated between preoperative tumour size and EOR.

The limitations of our study are its retrospective, non-randomized nature in a single tertiary referral centre, making it susceptible to selection bias for the management approaches during follow-up, although a prospective study would not be feasible in this setting. In addition, improved surgical technique and experience in endoscopic TSS over the 10 years (2009–2018) could also explain the improved EOR and preserved/ improved endocrine function noted in the latter part of the study period. Despite a relatively shorter follow-up, our findings are however comparable to longitudinal studies in other centres. Data from other UK series suggest 5-year tumour regrowth rates of 23–34% after surgery alone [25, 37] whilst we demonstrated a 18% relapse after a mean follow-up of 3 years. The advantages are a large cohort of nonselected subjects with non-functioning pituitary macroadenomas, reliable endocrine assessments by pituitary stimulation tests and accurate tumour volume measurements before and after surgery.

Importantly our data have shown dissociation between the endocrine and tumoral outcomes when related to adenoma size; tumour size being less important to endocrine outcomes than to the need for further intervention. This dissociation is further exemplified in our data by examining the cohort after stratification in to the first five, and second 5 years of surgical experience. These data showed increased EOR, and thus reduced risk of recurrence / progression, without significant adverse endocrine outcomes.

## Conclusions

In a large cohort of patients with NFPMA we have shown pre- and post-operative tumour volumes, as well as EOR do not correlate with the prevalence of pituitary hormone deficits, however, the post-operative residual volume is predictive of the requirement for further intervention to control the tumour mass. In the hands of an experienced neurosurgeon, total/ subtotal resection of pituitary adenoma is not a significant risk to pituitary function but may be protective of future invasive intervention, including radiotherapy and repeat surgery which have their own complications and risks to pituitary function.

## Data Availability

The datasets generated and/or analysed during the current study are property of Leeds Teaching Hospitals NHS Trust and cannot be shared publicly due to patient confidentiality purposes. Anonymized data are available from the corresponding author on reasonable request.

## Abbreviations

ACTH: Adrenocorticotropin
ADH: Antidiuretic hormone
DDAVP: Desmopressin
DI: Diabetes insipidus
EOR: Extent of resection
FSH: Follicle stimulating hormone
GH: Growth hormone
Gn: Gonadotropin
GST: Glucagon Stimulation Test
GTR: Gross total resection
HPA: Hypothalamo-pituitary-adrenal
ITT: Insulin tolerance test
IGF-1: Insulin-like growth factor 1
LH: Luteinising hormone
LTHT: Leeds Teaching Hospitals NHS Trust
MRI: Magnetic resonance imaging
NFPA: Non-functioning pituitary adenoma
NFPMA: Non-functioning pituitary macroadenoma
NTR: Near-total resection
RT: Radiotherapy
SRS: Stereotactic radiosurgery
SST: Short synacthen test
STR: Sub-total resection
T4: Thyroid hormone (thyroxine)
TSH: Thyroid stimulating hormone
TSS: Transsphenoidal surgery
